# On the Selective Laser Melting (SLM) of the AlSi10Mg Alloy: Process, Microstructure, and Mechanical Properties

**DOI:** 10.3390/ma10010076

**Published:** 2017-01-18

**Authors:** Francesco Trevisan, Flaviana Calignano, Massimo Lorusso, Jukka Pakkanen, Alberta Aversa, Elisa Paola Ambrosio, Mariangela Lombardi, Paolo Fino, Diego Manfredi

**Affiliations:** 1 DISAT, Department of Applied Science and Technology, Politecnico di Torino, Corso Duca degli Abruzzi 24, 10129 Torino, Italy; francesco.trevisan@polito.it (F.T.); jukka.pakkanen@polito.it (J.P.); alberta.aversa@polito.it (A.A.); mariangela.lombardi@polito.it (M.L.); paolo.fino@polito.it (P.F.); 2 Centre for Sustainable Future Technologies @PoliTo, Istituto Italiano di Tecnologia, Corso Trento 21, 10129 Torino, Italy; flaviana.calignano@iit.it (F.C.); massimo.lorusso@iit.it (M.L.); elisa.ambrosio@iit.it (E.P.A.)

**Keywords:** Additive Manufacturing (AM), Selective Laser Melting (SLM), AlSi10Mg, microstructure, mechanical properties

## Abstract

The aim of this review is to analyze and to summarize the state of the art of the processing of aluminum alloys, and in particular of the AlSi10Mg alloy, obtained by means of the Additive Manufacturing (AM) technique known as Selective Laser Melting (SLM). This process is gaining interest worldwide, thanks to the possibility of obtaining a freeform fabrication coupled with high mechanical properties related to a very fine microstructure. However, SLM is very complex, from a physical point of view, due to the interaction between a concentrated laser source and metallic powders, and to the extremely rapid melting and the subsequent fast solidification. The effects of the main process variables on the properties of the final parts are analyzed in this review: from the starting powder properties, such as shape and powder size distribution, to the main process parameters, such as laser power and speed, layer thickness, and scanning strategy. Furthermore, a detailed overview on the microstructure of the AlSi10Mg material, with the related tensile and fatigue properties of the final SLM parts, in some cases after different heat treatments, is presented.

## 1. Introduction

Additive Manufacturing (AM) technologies, combined with the development of new materials and the already available processes, have the potential of revolutionizing the way of perceiving the manufacturing of products. Generally speaking, the term AM refers to metals, while the term 3D printing refers to polymers. At the end of the 80s, industrial designers began to use 3D printing technologies to make prototypes of their projects in order to evaluate problems related to their form, fit, and functions, as well as their usability. In the late 90s and at the beginning of 2000s, 3D printing and AM technologies started to be employed in the production of final components, thanks to improved reliability and reproducibility of the processes, and in the case of metallic materials, thanks to the employment of powerful energy sources that were able to consolidate these materials. Today, AM has substituted certain conventional metal manufacturing processes, such as casting and forging, in some specific production routes, particularly in the aerospace and motor racing application fields. In fact, in these application fields its main benefits, such as the absence of constraints in the manufacturing design, shape freedom, high complexity of the components, combination of multiple parts into one part, production of functionally graded materials, reduced tooling requirements, and the possibility of production on demand, have been exploited [1,2,3].

In particular, over the last decade, an impressive development of the AM technology for metals named Selective Laser Melting (SLM) has taken place in order to produce complex shaped components in several structural alloys to meet the high demand requirements for applications in different fields, such as the aerospace, automotive, and biomedical sectors [4,5]. Moreover, it has been demonstrated, in recent literature, that SLM can also be used to fabricate metal matrix composites (MMCs) [6,7,8]. This goal can in part be reached through the development of new materials, with better properties than the same ones obtained by means of conventional processes, and in part through improvements in the control, accuracy, and reliability of the SLM process.

The SLM process, which has recently been defined as the laser powder bed fusion process, according to ISO/ASTM 52900 [9], and which is also known by the trade names Direct Metal Laser Sintering (DMLS) or LaserCUSING, directly produces homogenous metal objects, layer by layer, from 3D CAD data, by selectively melting fine layers of metal powder with a laser beam [10]. As SLM is an additive manufacturing technique, it allows structural parts to be built with the required strength and stiffness, but with considerably lighter weight than their conventionally manufactured counterparts [10,11,12,13]. For example, Jurg et al. [14] applied the SLM technology to the fabrication of a micro-lattice structure, adopted for the coolant jacket wall of a rocket engine, obtaining a net reduction of the overall dry mass of the engine, compared to previous designs. All these structures could be used to further reduce the airframe structure weight, guaranteeing at the same time the strength in resisting aerodynamic forces [15]. As observed by Klahn et al. [16], as far as SLM is concerned, only a few limitations to the design remain, and further development could be focused on improvements in the design to obtain a better performance. The development of specific application design guidelines should be taken into account to describe the best practices in order to resolve certain mechanical challenges [17,18,19].

As far as the lightweight materials that are currently available for SLM processing are concerned, the most frequently explored are titanium and aluminum alloys. The former ones, such as commercially pure Ti (CP-Ti) and Ti-6Al-4V (Ti64), have been in particular developed for biomedical applications, due to their high corrosion resistance and fatigue properties at room temperature [20,21]. On the other hand, the most frequently investigated Al alloys are Al–Si alloys, which represent 80% of aluminum casting alloys, thanks to their high fluidity, high weldability, good corrosion resistance, and low coefficient of thermal expansion. The binary Al-Si system is a eutectic system with about 12 wt % Si, as the eutectic composition is at 577 °C [22,23]. Al-Si alloys are defined as eutectic alloys when the Si is in the range 11–13 wt %, as hypoeutectic alloys when the Si is less than 11 wt % and as hypereutectic alloys when the Si is more than 13 wt % [24]. The strengthening of these alloys is generally possible, through the addition of other alloying elements, such as Mg and Cu, which make the Al-Si alloys hardenable either by means of a heat treatment, or by using rapid solidification techniques, in which the cooling rate is higher than 10^2^ K·s^−1^, such as melt spinning, which leads to a refinement of the microstructure [25]. However it is only possible to obtain ribbons or rods with melt spinning. In the SLM process, the interaction between the focused laser beam and the powders leads to an extremely high temperature gradient, with very high heating and cooling rates, as estimated in previous studies [26,27], thus a strong refinement is obtained in the eutectic Si phase and consequently a greater hardness and strength is measured for the final parts. Li et al. [27] performed a systematic investigation on the influence of the solution heat treatment on the eutectic microstructure and on the strength and ductility of an Al-12Si alloy produced by means of SLM. The results show that the size of the eutectic Si particles increases as the solution treatment time is increased. On the basis of a detailed Transmission Electron Microscopy (TEM) study, it was found that spherical Si particles, with a diameter of less than 100 nm, formed at the Al grain boundaries as a result of the extremely high cooling rate that is reached during SLM. Other interesting studies on Al–Si alloys have been summarized in a very comprehensive review by Olakanmi and co-workers [28]: the effects of the powders and the effect of the processing parameters on the densification mechanism and microstructural evolution in laser obtained samples have in particular been deeply analyzed.

As already stated, there is currently a great demand for Al–Si–Mg alloys for many applications, such as for motor racing, the automotive industry, and for aerospace and heat exchanger products, due to their high mechanical properties, like hardness and strength, in the heat treated state [29]. These alloys were traditionally used for lightweight and thin walled casting parts, and for any components with a complex geometry subjected to high loads [30]. Among these alloys, as reported by Sercombe in his review on Al and Al composites by SLM, the most frequently investigated is the AlSi10Mg alloy, which is similar to A360. Many studies have been performed on these alloys, considering such aspects as the starting powders up to the possible final applications of lightweight lattice structures [7,20,31,32]. The aim of the present review is to focus the attention on this Al hardenable alloy trying to draw up a comprehensive state-of-the-art portrait, and to correlate the effects of the main variables of the SLM process on the microstructural and mechanical characteristics of the alloy. Finally, this review also aspires to resume post process heat treatments developed for conventionally manufactured materials and applied on SLM materials, in order to analyze what effect these treatments would have on the overall mechanical behavior.

## 2. SLM Process

**Figure 1:**
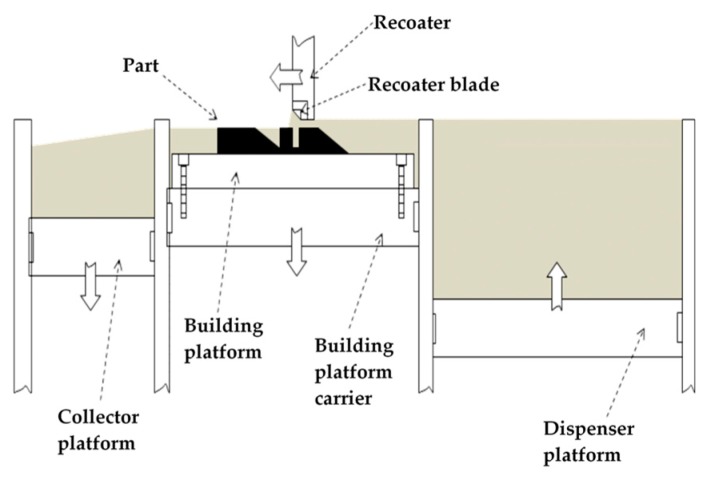
Schematic representation of the Selective Laser Melting (SLM) process.

The SLM process belongs to the class of powder bed fusion technologies known as selective laser sintering (SLS) and electron beam melting (EBM). The SLM process is schematically represented in Figure 1: a layer of powder (generally 20–60 μm thick) is spread over the building area, using a powder spreading system, which is commonly known as a recoater blade. Once the layer of powder is deposited on the building platform, which can be pre-heated, a laser source is directed onto the powder bed, and selectively fuses the material. The entire region of material subjected to the impinging heat energy is melted to a greater depth than the layer thickness. This type of consolidation is very effective in creating well-bonded, high density structures [33,34]. After completing a layer, the build platform is lowered by one layer of thickness, and a new layer of powder is laid, levelled, and melted. The process is repeated until a complete part is built. 

The SLM process involves a great variety of factors and variables that determine the final properties of the components, which are summarized in Table 1: they can be divided into two main categories: powder properties and process parameters. Each factor has a direct influence on the densification, microstructure, and mechanical properties of the final parts [7,35,36,37,38].

### 2.1. Effects of the Principal Powder Properties on SLM Parts

**Figure 2:**
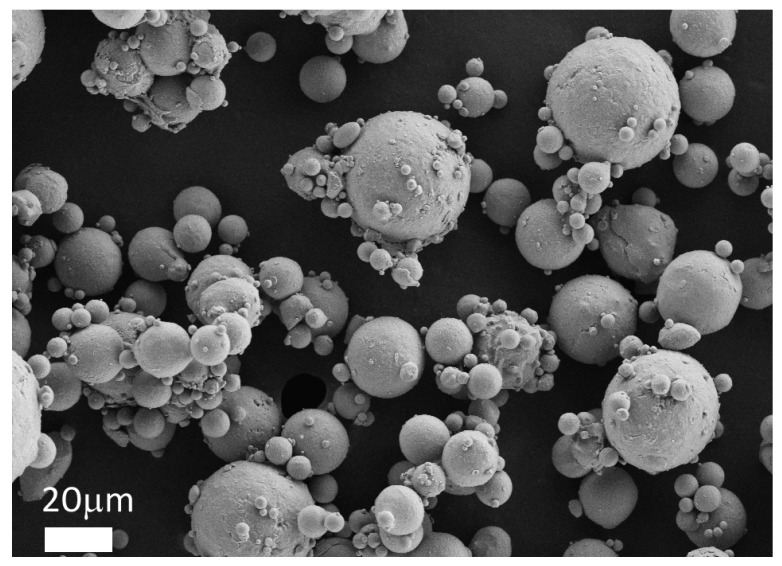
Field Emission Scanning Electron Microscopy (FESEM) image of gas atomized aluminum powders: the particles are spherical and their size varies between 1 and 30 μm.

SLM is based on the processing of metallic powders, and the properties of the fabricated parts therefore depend above all on the starting material that is used. Among the possible shapes that are suitable for metallic powders, the spherical one is preferred: it in fact allows a good flowability and high bed density. An example of a typical Al powder for an SLM process is shown in Figure 2: gas atomized powders are generally preferred for AM processes, because of their spherical shape. Water atomizing produces cheaper powders than gas atomizing, but they have a more irregular shape, and as a consequence, the flow time and packing density are reduced [39]. A good powder flowability is required to achieve constant thickness powder layers, that assure a uniform laser beam absorption in the building area [40]. Thicker regions of the powder bed may lead to insufficient re-melting depths in the previous layer, as well as favoring melt track instability effects [41].

Sercombe et al. [7] explored some of the inherent difficulties of working with aluminum alloys. They highlighted how the laser melting of aluminum poses several challenges for the production of high density components, because of the characteristics of the powder, which include stability of the oxide layer, poor flowability, high reflectivity, and high thermal conductivity. Spierings et al. [42] compared the densification and the mechanical strength of three stainless steel powders with different particle size distributions. They demonstrated how a certain amount of fine particles is needed in SLM processes in order to optimize the part properties (e.g., density, mechanical strength, surface roughness), while large particles determine the minimum layer thickness value, and are beneficial for the elongation at break of the final part. The size of the powder particles also influences the physical interactions between the powder and the laser beam. Fine powder particles in fact have a high surface energy, which in turn leads to superior densification kinetics, while large particles require higher incident laser density to be melted correctly [43]. As observed by Liu et al. [40], a possible direct correlation exists between the powder bed density and the part density: powders with a wider range of particle sizes providing a higher powder bed density and generating higher density parts under low laser energy intensities.

Another fundamental aspect is the chemical composition of the powders. They often have a high degree of contamination, which can be caused by moisture, organics, adsorbed gases, and oxide films that are present on the particle surface, due to their high surface area per unit volume: these contaminants not only inhibit the successful densification of the material, but also degrade the mechanical properties of the consolidated products [37,39,44]. Li et al. [45] investigated the chemical composition of the surface of A1-12Si alloy particles, before and after a drying treatment at 100 °C for 1 h. From the results, it appeared that adopting a drying treatment enabled the fabricated material density to be increased by more than 99%. Furthermore, through X-ray Photoelectron Spectroscopy (XPS) analysis, they found that the as-received powder surface contained Al metal and carbonate hydroxide (Al–O–CH_x_O). After drying, the presence of carbonate hydroxide decreased, while the amount of Al metal increased: the reduction in Al–O–CH_x_O was probably caused by the loss of moisture (H_2_O) from the powder surface. Finally, it is important to point out that Al reacts with moisture to produce Al oxide at temperatures of between 500 and 800 °C, while Al hydroxide is produced at temperatures over 800 °C. Both are known to form significantly during the SLM process, thus hindering the densification mechanism and favoring pore formation in the fabricated parts [37,43].

### 2.2. Effects of the Main Process Parameters on SLM Parts

As stated before, in the SLM process, a laser source is directed onto the powder bed, transferring heat energy to it and melting the material. In order to define the correct amount of energy that needs to be delivered to the powder bed, Simchi et al. [43] analyzed the combinations of parameters that influence the energy input during SLM. Assuming the material properties as fixed, the amount of energy transferred to the powder bed, during the laser irradiation-material interaction period, depends on the number of exposures and time between each exposure. The process parameters that have the most influence on the intensity and on the method adopted to deliver energy to a single layer of powder are: laser power (*P*), scanning speed (*v*), scan line spacing or hatching distance (*h*), and layer thickness (*t*). In order to evaluate the combined effect of these parameters, a factor called energy density (*ψ*) [46] was defined, with a Joule/millimetre^3^ (J/mm^3^) unit, according to Equation (1):

(1) *ψ* = *P*/(*v* × *h* × *t*)

Many authors have used this approach to study the SLM process of different materials [30,32,47]. For example, Meier et al. [47] focused on the processing map of 316 L stainless steel powders. Varying the process parameters, they found that the energy density range that was suitable to achieve an optimal combination of material density and surface quality was between 40 and 90 J/mm^3^, while an excessive delivery of the energy density resulted in melt pool instability and complications, such as balling. This phenomenon can be described as the sphereodization of the liquid melt pool during laser interaction with the powder bed [33,48].

On the other hand, taking into account that many commercial SLM machines keep the layer thickness value fixed, other authors [49,50,51] have not considered it for the process parameter optimization. As a result, they considered a surface energy density approach. Olakanmi et al. [39], for instance, studied the processing map for Al, Al-Mg, and Al-Si powders, investigating the different behavior of aluminum alloys on the basis of the energy inputs during SLM. The processing window was investigated by employing a laser power of between 20 and 240 W, and scanning speeds of between 20 and 250 mm/s, with a constant hatching distance of 0.1 mm. Four regions of densification behavior were identified for all the powders, and the findings can be summarized as follows:

- the region labelled “no marking” was related to a very low energy density (lower than 3.2 J/mm^2^), which did not permit an inter-particulate bonding between the particles;
- the “partial marking” region was characterized by an agglomerate network with a large amount of small, open and deep porosities, and can be related to the low energy density value that was used (between 3.3 and 10 J/mm^2^). The low energy input was not in fact able to generate an adequate liquid phase amount that would enable the full inter-bonding of the particles;
- dense parts (60%–80% density) were found in the “good consolidation” region, and were ascribed to the adoption of higher energy densities of between 12 and 30 J/mm^2^. The enhanced density was probably related to the higher powder bed temperature and lower viscosity of the melt pool of the processed powders, which facilitated the formation of an adequate amount of liquid phase, and this in turn promoted full melting;
- the occurrence of an “excessive balling” region was due to the high energy densities that were used to fabricate the parts (above 30 J/mm^2^), which favored the generation of an excessive liquid phase, and this in turn resulted in melt track instability and balling.

The hatching distance value has not been considered in the calculation of the energy density amount delivered to the powder bed in other studies [48,52,53,54,55,56]. In this way, the process window for the part processing was evaluated in terms of linear energy density. Starting from the consideration that the properties of manufactured parts depend to a great extent on each single laser-melted track and each single layer, Yadroitsev et al. [52], studied the effects of the scanning speed and laser power on the formation of single tracks for 316 L stainless steel, tool steel H13, copper alloy CuNi10, and superalloy Inconel 625. The results showed that the process has a stability zone where the track is continuous and an instability zone for low scanning speeds. The range of the optimal scanning speed is larger for higher laser power, and it narrows for high thermal conductivity materials.

The final SLM components showed different hardness and tensile properties from cast or wrought parts in similar alloys, and this was primarily due to the ultrafine microstructure, with its complex crystal growth directions, and to residual thermal stresses [45,57,58]. SLM parts generally present higher tensile strength, with anisotropy that depends on the building direction, than their cast counterparts [26]. The superior strength values that as-built components have are usually explained by adopting the Hall-Petch equation [59] reported in Equation (2):

(2) *σ*_0_ = *σ_i_* + *k*/*d*^1/2^

The equation highlights that the strength of the material (*σ*_0_) is given by the sum of the frictional stress (*σ_i_*) and a factor (*k*) times the inverse of the square root of the grain size (*d*). The local heating and cooling rates, during the melting of the powders, are very high (10^3^–10^8^ K·s^−1^) [45,60], and this leads to a non-equilibrium solidification process, with the development of ultrafine microstructures [61], extended solid solubility, and the possible formation of non-equilibrium phases. The relationship between the residual stress and the interfacial microstructure in a selective laser melted Al12Si/SiC composite was studied using confocal Raman microscopy by Li et al. [45]. The tensile stress in the SiC was found to be higher in the build direction (Z) than in the (X–Z) direction perpendicular to the build direction, while no such difference was observed in Si. The underlying reason for this difference was ascribed to the Gaussian distribution of the laser energy density and to stress relief through the interfacial reaction. Furthermore, the complex heat transfer conditions in the melt pool cause the preferential growth of grains, and this leads to a heterogeneous microstructure [26,59,60,61,62,63]. The presence of columnar grains, which are characteristic of some SLM materials, modifies the mechanical behavior of the material during the stress application, and thus generates anisotropy in the mechanical response.

**Figure 3:**
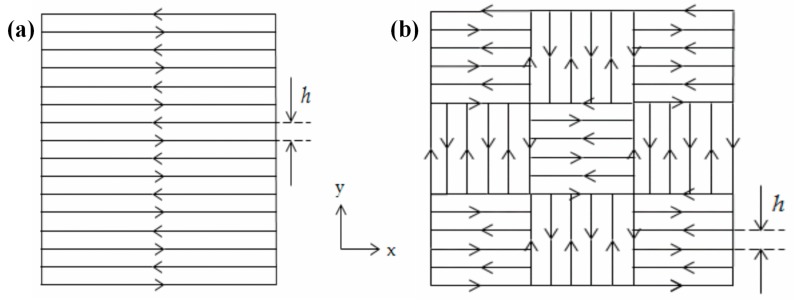
(**a**) Parallel scan line method; and (**b**) chessboard or island scanning strategy.

**Figure 4:**
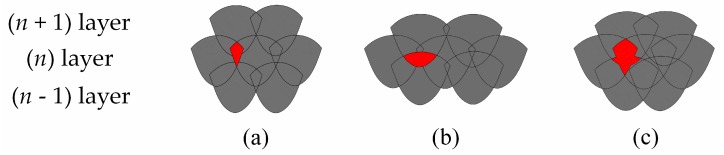
Three types of overlapping regimes under inter-layer stagger scanning strategy: (**a**) intra-layer; (**b**) inter-layer; and (**c**) mixed overlapping regime

The scanning strategy, which is the geometrical pattern that the laser beam follows during the hatching to melt and consolidate a section onto a layer, also influences the porosity and microstructure in SLM materials to a great extent [49,64,65,66]. Figure 3 shows two main scanning strategies. Moreover, adopting the correct scanning strategy helps to create final parts that are free from distortions, warping, porosities, and anisotropy. Guan et al. [67] studied the tensile properties of SLM 304 stainless steel, varying the hatch angle over 90°, 105°, 120°, 135°, and 150°, while keeping the other process parameters constant. From the results, they found that samples fabricated at the maximum interval number of layers, the test pieces fabricated at the hatch angle of 105°, have excellent yield strength and ultimate tensile strength due to a reduction in the residual stresses and anisotropy. Cheng et al. [68] developed a 3D sequentially-coupled finite element (FE) model to investigate the thermomechanical responses in the SLM process. The model was applied to different scanning strategies, and their effects on part temperature, stress, and deformation were evaluated. It was observed that the 45° line scanning case had the smallest deformation. Lu et al. [69] investigated the island size effect on the residual stress of SLM Inconel 718 parts. The results showed that a 5 × 5 mm^2^ island size sample had a lower residual stress than a sample with 7 × 7 mm^2^ or 3 × 3 mm^2^ island sizes. Su et al. [66] studied three different types of track overlapping regimes (intra-layer, inter-layer and mixed overlapping regimes) in order to select the best approach to improve fabrication efficiency as well as the relative density of the SLM process using 316 stainless steel (Figure 4). The results showed that an inter-layer overlapping regime could be reached when the track space was below 0.2 mm and the other parameters were kept constant; parts with the highest relative density were thus obtained. 

**Figure 5:**
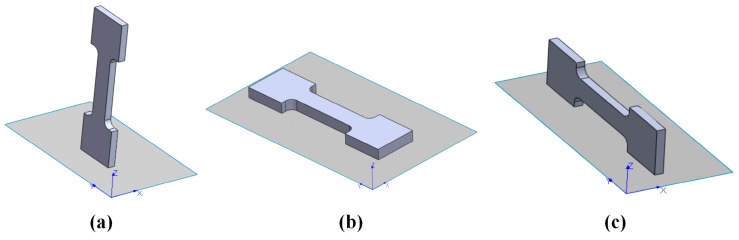
Examples of building orientations: (**a**) main direction parallel to the *xz*-plane; (**b**) main direction parallel to the *xy*-plane; and (**c**) main direction parallel to the *zx*-plane. The *z* axis in the picture indicates the building direction, while the *x* and *y* axes identify the building platform plane.

Another very influential and deeply investigated factor that affects the microstructure as well as the hardness values and tensile strengths of SLM materials is the building orientation of the part, which is graphically explained in Figure 5. The orientation can modify the microstructural evolution of the material, and can introduce anisotropy and defects [30,32,70,71]. Yadroitsev et al. [71] studied how the tensile properties of a specimen produced in Inconel 625 varied according to the different building orientations, underlining how the vertical specimens had a lower elastic modulus than the horizontal ones. The reason for this anisotropy was correlated to the higher number of defects, which were caused by a higher concentration of residual stresses, as was also observed in other studies [61].

**Figure 6:**
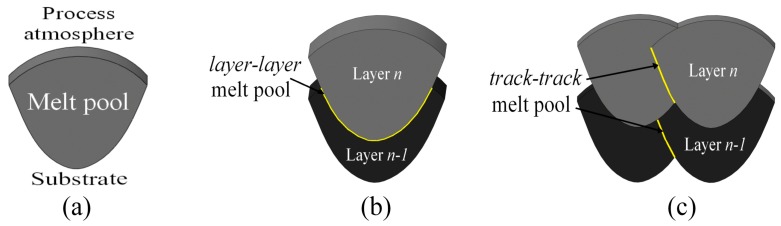
Schematic representation of different types of molten pool boundaries (MPBs): (**a**) single MPB; (**b**) “layer-layer” MPB; and (**c**) “track-track” MPB

However, the anisotropy in tensile properties found in the SLM parts has also been ascribed to the microstructural anisotropy caused by the local heat transfer condition, which can be determined by means of the scanning strategy [70]. Shifeng et al. [72] studied the influence of molten pool boundaries (MPBs) on the tensile properties of 316 L stainless steel SLM parts, built with different orientations. They identified two different MPB types in the component microstructure (Figure 6): “layer-layer” MPBs and “track-track” MPBs, generated by multi-layer and multi-track melt pool overlapping during the process, respectively. It was observed, from mechanical tests, that samples built along the *z* axis seemed to have lower tensile strength values and higher ductility than those built parallel to the *xy*-plane. The higher ductility could be related to the fact that the vertical samples have a larger number of slipping surfaces than the horizontal ones. In fact, when the vertical samples were loaded, slipping occurred simultaneously along both the “layer-layer” and “track-track” MPB surfaces, while in the case of horizontal samples, it only occurred along the “track-track” surfaces. 

Chlebus et al. [73] observed that the microstructural anisotropy of Inconel 718 influenced the fracture modes and the tensile properties, due to the different orientations of the columnar grains in relation to the different loading directions. The direction of the columnar grains depended on the heat flow that developed during the process, which is closely correlated to the building strategy that is adopted. 

The process atmosphere during SLM is another factor able to influence the amount and type of defects inside the material and therefore the mechanical behavior [28,37,74,75,76,77]. Fine metal powders are very sensitive to oxygen, due to their elevated surface areas, and the formation of an oxide layer alters the stability of the melt track during laser scanning, favoring balling phenomenon [28]. Moreover, when reactive powders, such as Al and Ti alloys, are considered, the O_2_ content should always be kept below 0.1% to avoid dangerous reactions. Wang et al. [74] investigated the influence of three different inert atmospheres, using high purity argon (Ar), nitrogen (N_2_), and helium (He), on the density and mechanical properties of Al-Si12 alloy parts. It appeared, from the results, that none of the different atmospheres significantly influenced the densification of the material. Only a slight difference was observed in the microstructure of parts produced with He: high porosity regions, composed of 50 μm diameter pores, were found in isolated areas. It appeared that the size of these areas did not affect the density measurement, but did influence the part ductility. Similar results were found in the study by Zhang et al. [78], who investigated how these three different protective atmospheres and hydrogen (H_2_), as a/the deoxidizer, mixed in different modes, influenced the porosity level of 316 L material. It was possible to note, from the results, that neither Ar nor N_2_ influenced the porosity amount when used in pure form, instead, when He or H_2_ was employed, the porosity increased by more than 10%. This phenomenon can be explained considering the different interactions between gases and the laser source, that is, in plasma conditions. The plasma generated from He or H_2_ is located at a higher position, with respect to the melt pool, than that generated by Ar or N_2_, because of the specific low gravity and different ionization energies. Once the plasma plume is far from the melted metal, the energy transport from the laser source can be obstructed, favoring the formation of porosities in the as-built SLM parts. Ferrar et al. [76] investigated the effect of an inert gas flow on the repeatability of the SLM process, and showed how this factor directly influenced the densification mechanism and the compression resistance of porous parts. The gas flow permitted the condensate material produced during laser melting to be removed, and this had a deleterious effect on the amount of energy absorbed during laser exposure. Masmoudi et al. [79] recently formulated a model to study the interaction between powder, laser and atmosphere during the SLM process: this model makes it possible to investigate how the argon gas pressure inside the process chamber could influence the melt metal behavior. It was found that lowering the overall pressure from 995 to 1 mbar, the solid laser track became less continuous and tended to disappear, leaving signs of a slight re-melting of the solid surface. The amount of evaporated material increased in a rarefied atmosphere (100 mbar), and the generated vapor expanded significantly in the atmosphere, in comparison to higher pressure conditions. In fact, an argon gas atmosphere permits oxidation to be reduced and evaporated material to be constrained on the surface of the material. 

## 3. AlSi10Mg Alloy

In recent years, numerous papers investigated the correlations between the SLM process parameters of AlSi10Mg and the related microstructures, corrosion resistance, residual porosity, hardness, tensile strength, yield strength, and elongation at break, in some cases after different heat treatments [32,49,80,81,82,83,84,85,86,87,88,89,90,91,92,93]. Table 2 summarizes some of these studies, highlighting the principal aims and the main findings.

**Figure 7:**
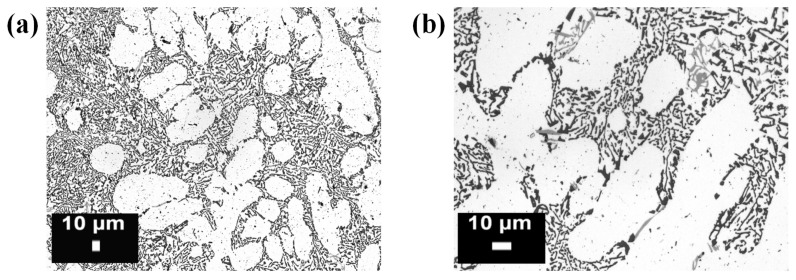
Optical micrographs of an AlSi10Mg alloy microstructure produced by means of casting (process,) at (**a**) low and (**b**) high magnifications: the white particles are composed of a α primary phase, while the matrix is composed of a eutectic α + Si phase (from the authors’ own unpublished work).

**Figure 8:**
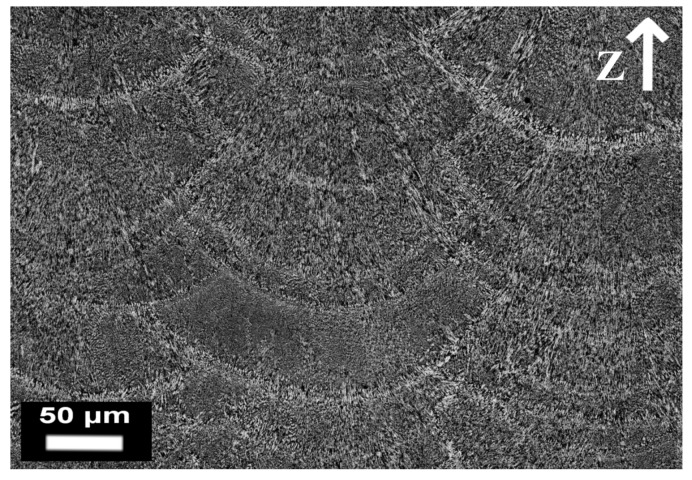
Optical micrographs of AlSi10Mg samples produced by means of Direct Metal Laser Sintering (DMLS) using a unidirectional scanning strategy (along the *x* axis). This strategy produces parallel scan tracks inside the material, and melt pools with an almost half cylindrical shape can be detected in a cross section parallel to the *zy* plane.

**Figure 9:**
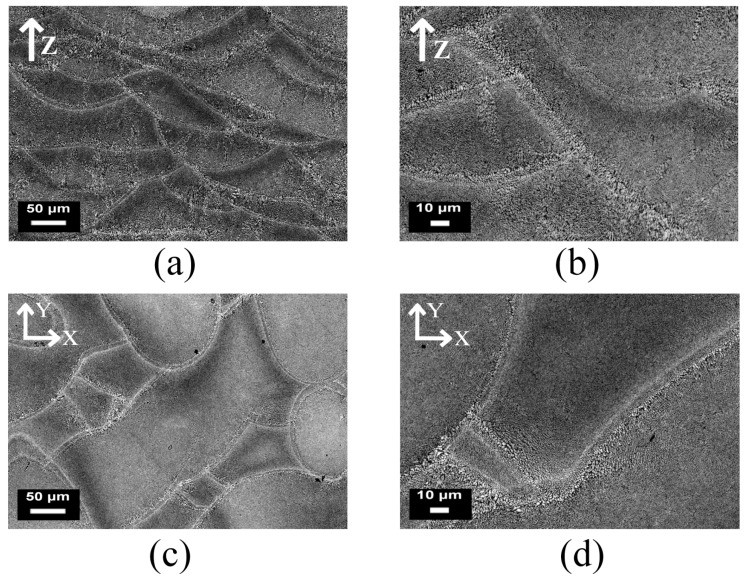
Optical micrographs of AlSi10Mg samples obtained by means of DMLS, built using a rotated scanning strategy (67° of rotation between subsequent layers), at different magnifications: (**a**,**b**) describe a vertical section of the part along the building direction (*z* axis); while (**c**,**d**) show the section parallel to the building plane (*xy*-plane).

In a traditional AlSi10Mg casting process, the solid solution of silicon in aluminum breaks down easily during slow cooling, and silicon precipitates in the form of relatively coarse particles, as shown in Figure 7: a continuous eutectic structure of Al and Si is generally obtained, along with dispersed primary α-Al. The SLM process, as previously stated, instead creates a unique micro- and macro-structure in the AlSi10Mg components, due to the repeated extremely fast melting and subsequent rapid cooling of the material. As described by Lam et al. [90], two different microstructures can be observed: a cellular-dendritic structure of α-Al and a network of the eutectic Si phase along the boundary surrounding the α-Al phase. The dimensions of the Al cellular dendrites, measured through TEM analysis, corresponded to 500–1000 nm, that is, much lower values than the ones of the cast parts. In a similar way, even though for another Al–Si alloy, Prashanth et al. [26] observed that the SLM process kinetically favored the solidification of α-Al into a cellular morphology, and the extended solubility of Si into Al. The residual amount of Si was preferentially located at the cellular boundaries, which had a thickness of about 200 nm. The overall macrostructure of the AlSi10Mg samples obtained by means of SLM, which was constituted by the overlapping of subsequent scan tracks, depended on the scanning strategy and process parameters that were adopted. Figure 8 and Figure 9 show examples of different macrostructures produced by means of different scanning strategies using a DMLS machine [80,93].

**Figure 10:**
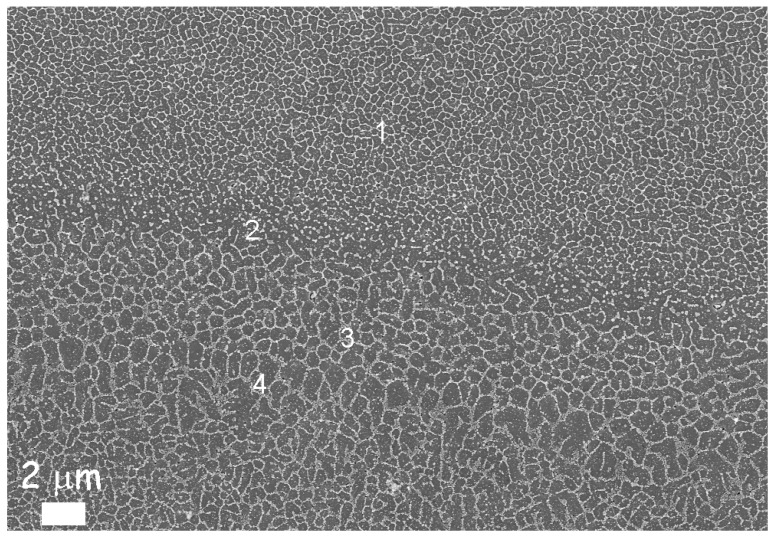
FESEM image of an AlSi10Mg alloy produced by means of the DMLS process in an as-built condition after etching with a Keller reagent: it is possible to observe the different sizes of the cellular-dendritic structures.

Another two important aspects involved in the melting process are directional cooling and rapid solidification, both of which have a profound influence on the microstructure of SLM parts. Manfredi et al. [80,93] investigated the AlSi10Mg microstructure, considering high magnifications, as illustrated in the FESEM micrograph of Figure 10. The area labelled as 2 and 3 corresponds to the heat affected zone of the adjacent melt pools: what is worth noting is the fine cellular-dendritic structure inside the melt pool (area 1), and the different size of the same structures due to thermal gradients (area 4). 

As described by Rosenthal et al. [88], the solidification in the SLM processing of an AlSi10Mg alloy depends on the thermal gradient (G) in the melt pool and on the growth rate (R). The growth rate could be modulated by changing the scanning speed and the angle among the direction of laser scan track and the growth direction of the solidified material. Lowering the R value at constant G values contributed to obtaining a stable planar consolidation front, while an increase in the growth rate induced the formation of cellular, and finally dendrite solidification morphologies. Multiplying G by R gives the cooling rate of the system: the higher the product, the finer the microstructure. Both the thermal gradient and the growth rate are at a maximum in the middle of the melt pool, and decrease slightly going towards the border: the growth rate reaches a zero value at the corner of the laser scan, where the laser path is perpendicular to the heat transfer [20]. As a consequence of modifying the G and R values, the fineness of the grains also varies.

Another effect of the directional solidification in the melt pool is the formation of a crystallographic texture inside the material. Thijs et al. [20] investigated the overall texture of AlSi10Mg parts produced by means of SLM, and the effects of different scanning strategies on the parts. It was evinced that when the cellular solidification mode was active, the preferential growth direction of the cells was along the 〈100〉 crystal direction, going towards the centreline of the melt pool. The thus obtained face centered cubic aluminum cells are decorated with a diamond-like silicon phase. This fine distribution leads to a high hardness of the SLM parts, of about 127 Hv0.5. Moreover, the fineness of the cells is seen to change from below 0.7 µm, at the melt pool border, to 0.4 µm in the middle of the scan track. Furthermore, on the basis of this study, it could be stated that a more anisotropic or isotropic part can be obtained according to which scanning strategy is applied.

In conventional manufacturing, the AlSi10Mg alloy is commonly strengthened through precipitation hardening [22], which consists of a solution heat treatment followed by quenching and artificial ageing. Adopting such a heat treatment on SLM materials modifies the microstructure to a great extent, as pointed out by Brandl et al. [85]. In their study, they showed how the as-built microstructure, constituted by cellular α-Al dendrites and interdendritic Si particles, was modified by a heat treatment, and that eutectic globular Si particles, homogeneously distributed inside the α-Al matrix, were obtained: all the microstructural differences, such as different grain size, heat affected zone, and melt pools, were eliminated. Li et al. [87] investigated the effect of different treatments, and highlighted how the microstructure became coarser when the solution temperature was increased from 450 to 550 °C, and in particular after artificial ageing (at 180 °C for 12 h). It was observed that when as-built SLM AlSi10Mg specimens were solution heat treated at 450 °C for 2 h, the mean Si particle size was less than 1 μm. When a 550 °C solution temperature was adopted, it rose to 4 μm, instead, when a subsequent artificial ageing was applied, the particles again became coarser, reaching a mean dimension of 5 μm. During the solution heat treatment and artificial ageing, the supersaturated Al phase rejected Si, which started to agglomerate in small particles. Aboulkhair et al. [89] recently established to what extent the SLM microstructure requires a different solution treatment duration from that of the casting materials. The fine microstructure of SLM AlSi10Mg required longer times than the cast one to be fully stabilized and homogenized: the precipitation behavior was completely different in a coarse microstructure from that of an ultrafine grained one.

The process and the adopted scanning strategy can deeply modify the microstructure and texture of the material [20,88], and they also have an important influence on the consolidation of the parts: the complexity of the melting and creation of defects inside the material has required a great deal of attention from both industry and research to study the correct process parameter window for SLM. One of the main aspects so far investigated is the extent of the porosity inside SLM materials, as this is a detrimental feature as far as the mechanical properties and fatigue life of aerospace components are concerned, since it compromises structural integrity and can favor premature and unexpected structural failure of the parts [61]. Kempen et al. [59] conducted an in-depth investigation of the correct choice of laser power and scanning speed with the aim of obtaining fully dense materials. Varying the laser power from 170 to 200 W and the scanning speed between 200 and 1400 mm/s, they were able to investigate the melting behavior of the powders adopting a laser energy per unit length approach. From the results, they established a process window in which it was possible to obtain 99% dense materials, while keeping the other process parameters fixed. The correct amount of energy in fact made it possible to avoid the entrapment of gases and an insufficient overlapping of the tracks, and consequently reduced the amount of porosity inside the material. 

**Figure 11:**
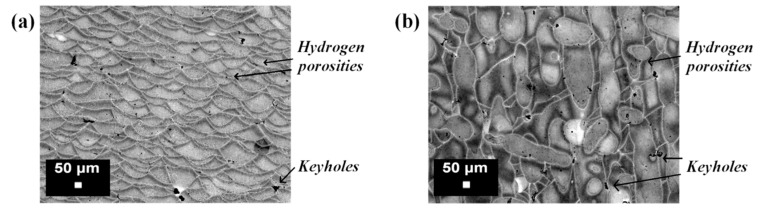
Optical images of an AlSi10Mg microstructure after Keller’s etching: (**a**) vertical section; (**b**) horizontal section. The metallurgical pores (hydrogen porosities) and the keyhole defects are indicated in the micrographs.

Considering hydrogen porosity, Weingarten et al. [44] investigated the formation of these pores in detail (Figure 11) during AlSi10Mg processing. The effect of a pre-drying treatment of the powder on the densification mechanism and porosity formation was studied, and it was established that a heat treatment of the powder bed at 200 °C, before the melting process, led to a reduction in hydrogen porosity of approximately 50%. The drying treatment in fact reduced the amount of moisture on the powder surface, an aspect that is related directly to the porosity formation during melting [43,45].

**Figure 12:**
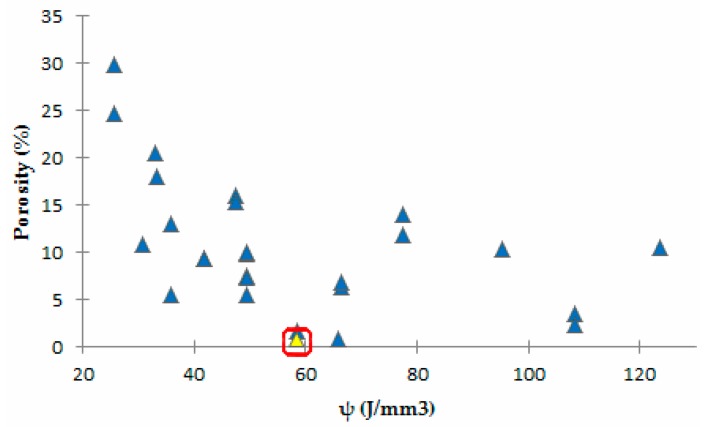
Porosity variation versus volumetric energy density. The highlighted point represents the lowest porosity level obtained for the SLM processing of AlSi10Mg powders.

In another study, Aboulkhair et al. [49] investigated the effects of different process parameters on the formation of defects inside the microstructure of AlSi10Mg samples. The metallurgical pores formed predominantly in samples built with a low scanning speed (250 mm/s), while keyholes were found for higher speed rates (higher than 500 mm/s). The reason for this was attributed to the too high energy amount delivered to the material. With a high scanning rate and low laser power values, balling altered the solidification behavior and modified the consolidation of the layers. On the other hand, adopting low scanning speeds and high laser power induced an increase in the energy per unit length, which in turn induced melt instability. Furthermore, the distance between each scan track influenced the consolidation behavior of the melt: a lack of overlapping between scan tracks implied a notable increase in the porosity level [49]. Read et al. [32] used a statistical approach to evaluate the influence of process parameters on the porosity of an AlSi10Mg alloy produced by means of the SLM process. Using ANOVA, they were able to delineate the porosity response as a function of the laser power, hatching distance, scanning speed, and scanning strategy. As expected, the main influence on the porosity level was attributed to the first three parameters, as also shown in previous studies [28,35,39]. Since the laser power, scan speed, and hatching distance could individually influence and control the energy input, it was conceivable that the porosity level could be lowered by modifying one of these parameters, changing their values in the range delimiting the process window. In fact, when the laser power was kept fixed, it appeared that lowering the hatching distance value eliminated the effect of the scan speed on the formation of porosity. By working within the process parameter window, it was found that the amount of energy input necessary to reduce the porosity to the minimum value was approximately 60 J/mm^3^ (Figure 12), which is comparable with the threshold limit found in the research conducted by Olakanmi on Al, Al–Si, and Al–Mg alloys [39]. 

All of the above described studies concluded that the process parameters do in fact influence the porosity level, because of the direct effect they have on the energy amount transferred to the powder bed. Energy density values (per length [59], per area or per volume [32]) offer an indication of the process window limits, but it is necessary to carry out an accurate study of the influence of the process parameters on the porosity formation to establish their correct values for an optimized SLM process.

**Figure 13:**
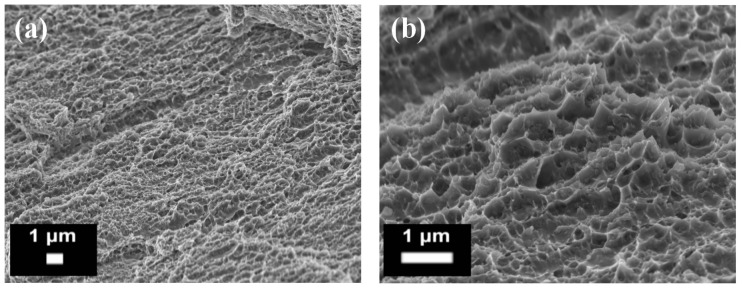
Example of a fracture surface of an AlSi10Mg alloy obtained by means of SLM observed by FESEM, at (**a**) low; and (**b**) high magnifications.

**Figure 14:**
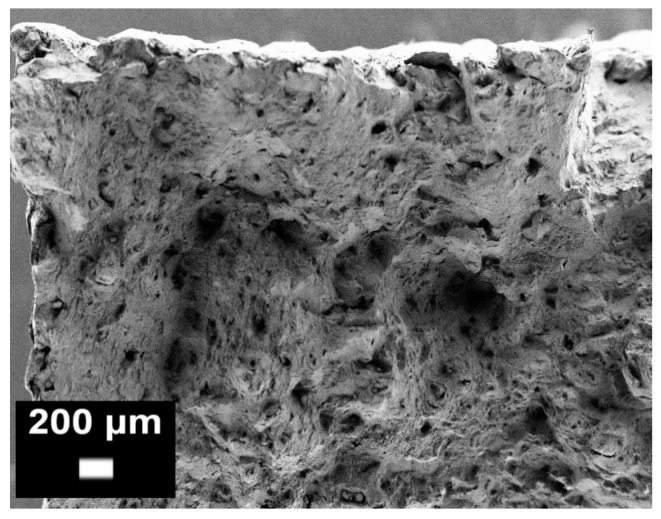
Example of an SLM AlSi10Mg fracture surface after a tensile test observed by FESEM. The presence of significant micro-porosity on the fracture surface can easily be evinced (from the authors’ own unpublished work).

**Figure 15:**
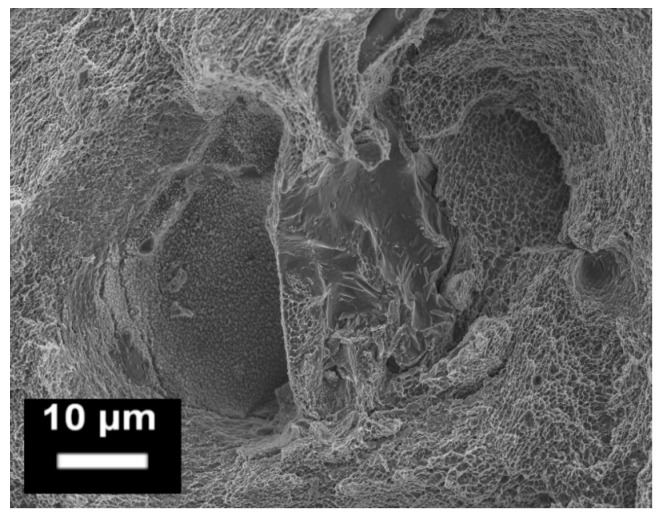
Example of oxide layers on the fracture surface of an SLM AlSi10Mg tensile specimen observed by FESEM. The central smooth area represents a fragile rupture region, and is different from the surrounding fine dimple regions, which are characterized by ductile fractures (from the authors’ own unpublished work).

**Figure 16:**
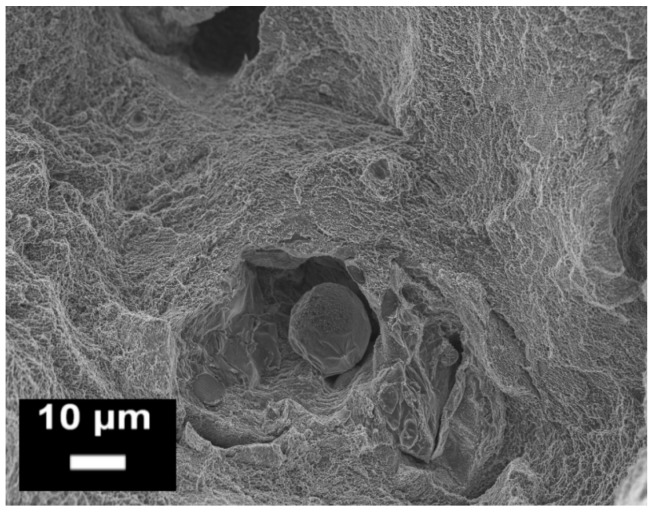
Example of an un-melted powder particle inside an SLM AlSi10Mg tensile sample observed at by FESEM (from the authors’ own unpublished work).

As far as the mechanical behavior of SLM parts is concerned, it has been shown that they have a higher tensile strength and, at the same time, a comparable ductility and a lower fatigue life than parts produced via a traditional manufacturing process. The reason why AlSi10Mg parts produced by SLM present higher hardness, and tensile strength than traditionally cast ones can be explained considering three main factors [90]: the ultrafine-grained microstructure, which favors grain boundary strengthening, the alloying elements, which are responsible for solid solution strengthening, and the strengthening given by the interactions of dislocations. Buchbinder et al. [31] tested AlSi10Mg alloy samples produced by means of SLM using two different building parameter sets: 240 W laser power and a 500 mm/s scanning distance for the first one, 960 W laser power and a 1000 mm/s speed for the other. The ultimate tensile and yield strengths appeared to be independent of the laser power, and similar values were obtained in both cases (UTS = 400–450 MPa; YS = 210–240 MPa). The elongation at break of samples produced with high laser power was 25% higher than in the case of low power. Furthermore, it was described how three main aspects should be taken into account regarding the elongation at break anisotropy in an SLM process: defects and pore presence, grain orientation and texture, as well as interfaces between melt tracks and layers. Contrasting results were found by Rosenthal et al. [88], who reported a higher elongation at break for specimens built horizontally than those built vertically. The reason for this was found after an analysis of the fracture surfaces: the vertically built specimens exhibited a predominantly ductile failure located between weakly bonded layers, while fracturing occurred within each layer in the horizontally built ones. Kempen et al. [82] showed similar results, and obtained a mean elongation at break of 5.55% for horizontal samples and 3.47% for vertical ones. Large pores, which were responsible for fracture initiation during the tensile test, became the preferential sites for inhomogeneous deformation at a high stress level (around 395 MPa), and consequently for cracking initiation. These defects were found to be more numerous in vertical samples than in horizontal ones. Read et al. [32], analyzing fracture surfaces, found the presence of thick oxide layers on the particles: these un-bonded regions gave rise to large cracks during failure. These un-bonded areas appeared flat or faceted, in comparison to the fracture regions where ductile deformation occurred [35]. Examples of AlSi10Mg SLM fracture surfaces are reported in Figure 13, while the typical defects of SLM are illustrated in Figure 14, Figure 15 and Figure 16: porosities (Figure 14), oxide layers (Figure 15), and un-melted powders (Figure 16) generally have a marked detrimental effect on the hardness and tensile properties of parts [32,80].

**Figure 17:**
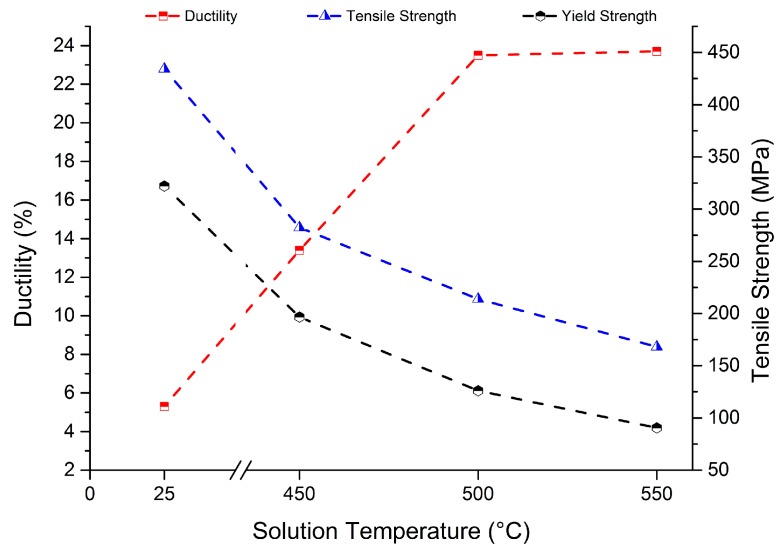
Tensile properties of AlSi10Mg alloy specimens produced by means of SLM, as-built and after solution heat treatments at different temperatures. The post process solution heat treatment was conducted for 2 h and was followed by water quenching at room temperature.

As previously mentioned, the entire SLM process introduces high thermal residual stresses inside the material that generally provoke distortion, warping, and alteration of the mechanical properties of the fabricated parts. In order to reduce the amount of residual stress, while maintaining good dimensional and physical stability, stress relieving is commonly performed on the SLM parts after the AM process. A stress relieving treatment reduces the tensile strength of the as-built parts to a great extent, and increases the ductility level. Mertens et al. [92] studied the effects of a stress relieving treatment at 250 °C for 2 h on AlSi10Mg samples: the elongation at break exhibited an increase of 80%, while slight decreases of 12% and 2% were recorded for the yield and ultimate tensile strengths, respectively. The importance of a post process heat treatment appears fundamental to improve the ductility behavior of SLM as-built parts, while maintaining the high tensile properties, and its definition has already been studied in literature [45,80,92]. Li et al. [87] have recently investigated the influence of solution and artificial ageing on SLM-produced AlSi10Mg alloy samples. From the results, which are schematically summarized in Figure 17, it appears that each solution heat treatment reduces the tensile strength to a great extent, compared to the high values of as-built parts (UTS = 434 MPa). It was in fact found that the higher the temperature, the lower the tensile properties, till minimum ultimate and yield strength values of 168 and 90 MPa, respectively, were recorded for 550 °C solution heat treated samples. At the same time, the ductility of the specimens passed from 5.3% to a maximum of 23.7%. An ageing treatment did not enhance the low tensile properties after solution and quenching treatments: the maximum tensile strength remained at around 200 MPa, while the elongation at break remained at 23%.

**Figure 18:**
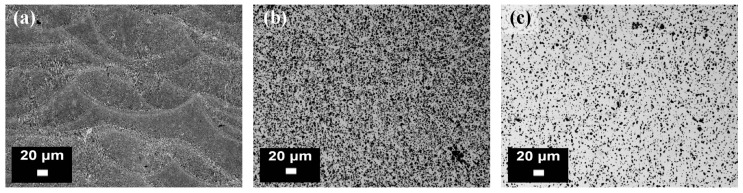
Optical images of an AlSi10Mg alloy produced by means of DMLS and heat treated: (**a**) after a stress relieving at 300 °C for 2 h; (**b**) after a T4 treatment (**c**) after a T6 treatment (from the authors’ own unpublished work).

**Figure 19:**
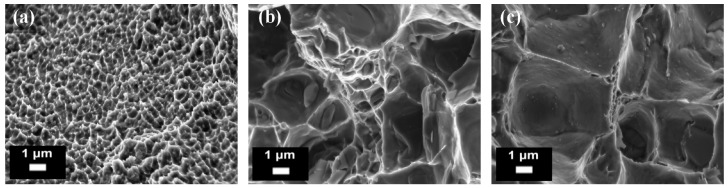
FESEM micrographs of fracture surfaces of AlSi10Mg samples produced by means of DMLS and heat treated: (**a**) as-built conditions; (**b**) after a T4 treatment; (**c**) after a T6 treatment (from the authors’ own unpublished work).

Mertens et al. [92] instead studied the effects of different ageing temperatures and times on the hardness and tensile properties of solution heat treated (510 °C for 6 h) and water quenched AlSi10Mg parts. From the results, it was established that the best-compromise was an ageing treatment at 170 °C for 4 h: in comparison to the as-built conditions, the tensile strength decreased by 13%, but the yield strength and the elongation at break increased by 30% and 220%, respectively. The microstructure was also affected a great deal by the post-processing treatment: the silicon lamella structures were in fact substituted by globular silicon precipitates. Manfredi et al. [80] studied the effects of a T4 treatment (solution treatment at 530 °C for 5 h, followed by quenching in water, and then by room temperature ageing for at least two weeks) and of a T6 treatment (solution treatment at 530 °C for 5 h, followed by water quenching, and then by artificially ageing at 160 °C for 12 h). The optical micrographs of the microstructure of the parts are reported in Figure 18 after each heat treatment, while the FESEM micrographs of the fracture surface are illustrated in Figure 19: these images highlight the coarsening effects of the heat treatments on the microstructure, which in turn become more isotropic. 

When considering high demanding applications, an important property is the fatigue resistance of the components. Brandl et al. [85] investigated the high cycle fatigue (HCF) properties of an AlSi10Mg alloy: un-notched (stress concentration factor, K_t_, equal to 1) samples were tested with a test frequency of 108 Hz, adopting a tension-tension mode with a stress ratio (R) equal to 0.1 (corresponding to a tension-tension cycle in which the minimum stress is equal to 1/10 of the maximum one). The influence of the building platform temperature and T6 treatment (solution treatment at 525 °C for 6 h, followed by quenching and ageing at 165 °C for 7 h) on the endurance limit was analyzed, and it was pointed out how the combination of 300 °C platform heating and the peak hardening condition was able to increase the fatigue resistance of the material. In fact, both factors helped to homogenize the properties of the parts, and in particular the T6 treatment, which altered the as-built microstructure by removing heat affect zones (HAZ), thus favoring the sphereodization of interdendritic eutectic Si particles (microstructural difference) and reducing the crack initiation and/or propagation time. Furthermore, it was also highlighted how porosity had the most detrimental effects on the fatigue properties, especially when the pore size exceeded a certain value. Similar conclusions were presented by Mower and Long [63], concerning the fatigue properties of AlSi10Mg, evaluated through fully-reversed bending tests (frequency equal to 20–25 Hz): for any prescribed lifetime, the maximum endurance limit of the SLM specimens (as-built conditions) was 30% lower than the reference wrought 6061 aluminum alloy specimens. This different behavior was explained by the presence of defects and porosities inside the SLM material, which acted as stress concentration sites that favored crack initiation and propagation. 

## 4. Conclusions

AM technologies are revolutionizing the industrial manufacturing world, by allowing the final products to be designed innovatively and efficiently in several types of applications, ranging from the biomedical to the aeronautical and aerospace fields. The reduction in weight, in waste material, and in the design limitations favor economic and scientific interest in the development of these technologies. Among the AM used for metals, Selective Laser Melting (SLM) has been developed for a wide range of alloys since the introduction of commercial systems about a decade ago. The final parts obtained from SLM present higher tensile strength than traditional manufacturing products: the specific metallurgical conditions that are present during the process, such as rapid solidification, directional heat flux, and temperature gradient, allow ultrafine microstructures to be created inside the final parts. Nevertheless, metallurgical defects, such as gas entrapment porosities, oxide layers, and un-melted material can easily be generated during the process, and they reduce the performances of the final components. In order to produce high-value final products, the process parameters must be optimized correctly for each metal powder system. An extensive number of researches on SLM technology have been carried out in recent years on aluminum alloys in order to have a complete comprehension of the process. In this review, an extensive analysis has been made of the main SLM process parameters and variables, focusing on Al alloys, and in particular on the hardenable AlSi10Mg alloy. The state of the actual research on the microstructural and related mechanical behavior of samples fabricated with this alloy has been described, considering also the fundamental post-heat treatments. The use of standard metallurgy heat treatments shows that they do not lead to the usual results. SLM produced parts need to be treated differently from bulk alloy parts. The highest hardness, yield, and ultimate tensile strengths were obtained in the as-built conditions. However, for industrial production purposes, it is fundamental to reduce the residual stresses generated during the SLM process in order to guarantee dimensional stability and reproducibility. The need for future studies on how to define new heat treatments for this hardenable alloy has clearly emerged.

## Figures and Tables

**Table 1:** Selective Laser Melting (SLM) variables.

Powder Properties	Process Parameters	
Particle shape	Laser power	Layer thickness
Particle size and distribution	Scanning speed	Scanning strategy
Chemical composition	Hatching distance	Building orientation
Thermal conductivity	Protective atmosphere	Gas flow
Melting temperature	Laser beam radius	Bed temperature
Absorptivity/reflectivity	Laser type	-

**Table 2:** The main recent studies focused on AlSi10Mg alloy processed by SLM.

Principal/Main Aims of the Research	Findings	Ref.
Microstructure	Fine microstructure with submicron-sized cells	[20]
	High hardness (127 ± 3 Hv0.5).	
	Morphological and crystallographic texture.	
Porosity, tensile and creep responses	Better strength and elongation properties than die cast Al-alloys of similar composition.	[32]
	Creep results showed better rupture life than cast alloy	
Hydrogen porosity	The moisture on the powder particle surface and the dissolved hydrogen in the powder materials lead to nucleation and the growth of hydrogen pores in the melt pool that can be reduced by drying the powder. The hydrogen pores can be affected during the process by different parameters, such as the time between the melting and the solidification.	[44]
Porosity	A compromise between the process parameters and scan strategies can produce parts with a density of 99.8%	[49]
Microstructure, high cycle fatigue, fracture	High fatigue resistance.	[85]
	The combination of 300 °C platform heating and peak-hardening (T6) increases the fatigue resistance and neutralizes the differences in fatigue life for the 0°, 45° and 90° directions	
Heat treatment	The tensile strength decreases from 434.25 ± 10.7 MPa for the as-built samples to 168.11 ± 2.4 MPa, while the elongation at break increases remarkably from 5.3% ± 0.22% to 23.7% ± 0.84% when the as-built sample is solution-treated at 550 °C for 2 h.	[87]
Precipitation hardening	The duration of the SHT (Solution Heat Treatment) influences the ageing response. A fine microstructure requires a longer SHT to stabilize the microstructure and enhance the mechanical response, with and without ageing.	[89]
Phase analysis, microstructure characterization	A certain amount of Si dissolved in the Al matrix to form cellular-dendritic α-Al phase (cells of about 500 nm). An Mg_2_Si dispersoid hardening phase formed during the SLM process. The network features along the boundary of the Al phase were identified to be a eutectic Al/Si phase. Very fine grainy features of a nanometric scale were observed within this phase, with dimensions of less than 5 nm.	[90]
Microstructure, heat treatments, hardness, and tensile properties	A fine microstructure with submicron-sized cells	[80,93]
	High hardness, Yield, and Ultimate tensile strength	
	Effects of T2, T4, and T6 heat treatments	
Corrosion resistance	Preferential dissolution of α-Al at the border of the laser scan tracks	[81,83,84]
	Modification of the surface, by means of shot peening or polishing, increases the pitting potential and reduces the corrosion rate	
